# Adjuvant Analgesics in Acute Pain – Evaluation of Efficacy

**DOI:** 10.1007/s11916-024-01276-w

**Published:** 2024-06-12

**Authors:** Isabelle Kummer, Andreas Lüthi, Gabriela Klingler, Lukas Andereggen, Richard D. Urman, Markus M. Luedi, Andrea Stieger

**Affiliations:** 1 Department of Anesthesiology, Rescue- and Pain Medicine, Cantonal Hospital of St. Gallen, St. Gallen, Switzerland; 2 Department of Neurosurgery, Cantonal Hospital of Aarau, Aarau, Switzerland; 3 Faculty of Medicine, University of Bern, Bern, Switzerland; 4 Department of Anesthesiology, The Ohio State University, Columbus, OH 43210 USA; 5 Department of Anesthesiology and Pain Medicine, Inselspital, Bern University Hospital, University of Bern, Bern, Switzerland

**Keywords:** Adjuvant analgesics, Co-analgesics, Perioperative pain management, Ketamine, Magnesium, α2-agonists, Dexamethasone, Intravenous lidocaine

## Abstract

**Purpose of the Review:**

Acute postoperative pain impacts a significant number of patients and is associated with various complications, such as a higher occurrence of chronic postsurgical pain as well as increased morbidity and mortality.

**Recent Findings:**

Opioids are often used to manage severe pain, but they come with serious adverse effects, such as sedation, respiratory depression, postoperative nausea and vomiting, and impaired bowel function. Therefore, most enhanced recovery after surgery protocols promote multimodal analgesia, which includes adjuvant analgesics, to provide optimal pain control. In this article, we aim to offer a comprehensive review of the contemporary literature on adjuvant analgesics in the management of acute pain, especially in the perioperative setting.

**Summary:**

Adjuvant analgesics have proven efficacy in treating postoperative pain and reducing need for opioids. While ketamine is an established option for opioid-dependent patients, magnesium and α2-agonists have, in addition to their analgetic effect, the potential to attenuate hemodynamic responses, which make them especially useful in painful laparoscopic procedures. Furthermore, α2-agonists and dexamethasone can extend the analgesic effect of regional anesthesia techniques. However, findings for lidocaine remain inconclusive.

## Background

The International Association for the Study of Pain (IASP) defines pain as an *“unpleasant sensory and emotional experience associated with, or resembling that associated with, actual or potential tissue damage”* [1]. Pain plays an integral part in the medical care of patients. Acute pain accounts for up to 70% of visits to the emergency departments which makes it one of the most common reasons for patients seeking medical care [2]. Besides, acute pain is of equal importance in the perioperative setting. Several studies have indicated that approximately 50% of patients suffer from moderate to severe pain within the initial 24 h following surgery [3–5].

Acute postoperative pain does not only affect patient satisfaction but might also lead to chronic postsurgical pain: several studies detected a correlation between the intensity of acute pain in the postoperative period and the emergence of chronic postsurgical pain [6–8]. In addition, inadequate analgesia can result in various other complications such as an increased incidence of pulmonary or cardiac complications and even increased morbidity and mortality [4]. Risk factors contributing to heightened pain levels after surgery include female gender and young age [3]. Preoperative quantitative sensory testing may anticipate postoperative pain in patients undergoing elective procedures and may help to find patients prone to experience severe pain after surgery [9].

Therefore, it is essential to effectively manage acute pain [10–14]. Although opioids are often used in the management of postoperative pain due to their effectiveness in alleviating even severe pain, their side effects, such as sedation, respiratory depression, postoperative nausea and vomiting (PONV) and impaired bowel function can extend the duration of hospitalization [15]. Furthermore, overuse of opioids can exacerbate opioid dependence in susceptible patients, even with short-term use [16]. Hence, most enhanced recovery after surgery (ERAS) protocols, as for example in cardiac surgery [17], promote multimodal analgesia, which incorporates various methods of pain management to attain effective pain relief while mitigating opioid-related side effects. [18].

Adjuvant analgesics, also known as co-analgesics, are important in the treatment of chronic pain: they may enhance the analgesic effect of conventional analgesics or have independent analgesic activity of their own in certain conditions such as neuropathic pain. When added to an opioid therapy, they can enhance pain relief, address refractory pain and lower opioid doses, which reduces opioid’s adverse effects [19]. However, their contribution to perioperative pain management remains elusive.

This narrative review aims to offer an overview of the current literature on the role of adjuvant analgesics in the multi-modality management of acute pain, in particular within the perioperative context. We seek to explore the efficacy of adjuvant analgesics, both for parenteral administration and as adjuncts to regional anesthesia, where appropriate.

## N-methyl-D-aspartate (NMDA) Receptor Antagonists

The amino acid glutamate is the most important excitatory neurotransmitter within the central nervous system. Glutamate activates NMDA receptors in the spinal cord causing the spinal cord neuron to become more responsive to its inputs, which eventually leads to central sensitization [20]. Therefore, NMDA receptor antagonists have been pivotal in preventing hyperalgesia and in managing chronic pain. However, as multimodal analgesia gains importance, NMDA receptor antagonists are also becoming integral in the treatment of acute pain, as their blockade is believed to enhance the efficacy of opioids [21].

The most important NMDA receptor antagonists used as adjuvant analgesics in acute pain management are ketamine and magnesium sulfate.

### Ketamine

Ketamine is a phencyclidine derivative and dissociative anesthetic agent. It exerts its analgesic effect via its reversible antagonism on the NMDA receptor, although it has some effect on the μ-opioid, muscarinic, monoaminergic and γ-aminobutyric acid receptors as well [22].

In the perioperative setting, ketamine is mainly used as an adjuvant analgesic in painful procedures, including abdominal, thoracic, and major orthopedic surgery, as well as in opioid-tolerant or -dependent patients presenting for surgery [23]. Several smaller randomized controlled trials (RCT) suggest that subanesthetic doses of ketamine reduce postoperative pain scores and the need for opioids [24–26]. A systematic review conducted by Laskowski et al. demonstrated a significant decrease in total opioid consumption and an increase in the interval until the first analgesic was administered in patients receiving intravenous ketamine across all studies. The greatest efficacy was found in painful procedures like thoracic and upper abdominal, where the greatest reduction in opioid use was seen, as well as in lower abdominal and major orthopedic surgeries [21].

There is limited evidence for opioid-dependent patients, based on a few RCTs with conflicting results: One RCT by Loftus et al. showed that ketamine decreased opioid consumption at 48 h postoperatively as well as pain intensity in the postanesthesia-care unit (PACU) in opioid-dependant patients undergoing back surgery [27]. However, another study in the same patient population showed no benefit [28].

Based on the existing evidence, the consensus guidelines on the use of intravenous ketamine infusions for acute pain management from the American Society of Regional Anesthesia and Pain Medicine, the American Academy of Pain Medicine and the American Society of Anesthesiologists, published by Schwenk et al. in 2018, suggest that “subanesthetic ketamine infusions should be considered for patients undergoing painful surgery and ketamine may be considered for opioid-dependent or opioid-tolerant patients undergoing surgery” [23].

Apart from its role in the perioperative setting, ketamine has been used in prehospital trauma care for a long time. In the trauma setting, it is especially useful as it has analgesic features but also affects the sympathetic nervous system and increases the average heart rate and blood pressure [29].

#### Adverse Events

Most studies on ketamine use for acute pain management provide some insight on adverse events. The most reported adverse events of ketamine use are nausea, vomiting, vivid dreams, and hallucinations. However, in most studies, the incidence of adverse events is only slightly higher compared with placebo [21, 30].

According to the guidelines by Schwenk et al. ketamine usage is discouraged in patients with poorly managed cardiovascular disease, psychosis, or severe hepatic disease as well as in pregnant women [23].

### Magnesium Sulfate

The analgesic effects of magnesium are believed to be associated with the control of calcium influx into the cell [31] and its antagonistic effect on the NMDA receptor in the central nervous system [32, 33].

Several studies demonstrated that administering magnesium sulfate perioperatively reduced postoperative opioid consumption as well as pain scores at rest and on movement, whether it was given as a continuous infusion or as a single bolus dose [34–36].

The rising prevalence of laparoscopic techniques has prompted investigations into the effects of magnesium sulfate on cardiovascular reactions. It has been shown that the perioperative administration of magnesium sulfate was associated with a diminished hemodynamic response, characterized by decreased blood pressure and heart rate, following the induction of pneumoperitoneum [37–39]. They found that patients receiving high doses of magnesium sulfate had significantly lower systemic vascular resistance, mean arterial blood pressure and central venous pressure while the cardiac output was significantly increased compared to the control group that was given normal saline [39]. Furthermore, the group administered high doses of magnesium sulfate experienced better pain control, as indicated by a lower visual analogue scale (VAS) score.

Therefore, magnesium sulfate could serve as a crucial component in the perioperative period, not only to alleviate pain but also to attenuate hemodynamic responses to pneumoperitoneum, making it a good adjuvant for (painful) laparoscopic surgeries.

Apart from its use in the perioperative setting, magnesium sulfate might be beneficial in the pain management of patients with dysmenorrhea [40] and migraine [41]. However, high quality evidence is missing and there is the need for further randomized controlled trials evaluating the effect of magnesium sulfate in this regard.

#### Adverse Events

Magnesium sulfate has a broad therapeutic index. Known adverse events of magnesium sulfate include prolongation of neuromuscular blockade after administration of non-depolarizing neuromuscular blocking agents [42, 43], sedation [44], dizziness and rarely respiratory depression [45]. Serious cardiovascular events have been described in relation with iatrogenic overdose [46]. The systematic review of Albrecht et al. showed that bradycardia was common after magnesium administration but there were no reports of persistent hemodynamic instability although doses as high as 23.5 g over a period of 24 h have been administered in one study included in the review. No difference in the occurrence of sedation or hypotension was noted. However, the incidence of adverse events might be underestimated as only six studies evaluated the incidence of hypotension and bradycardia and only two studies evaluated the incidence of sedation [35].

## α2 Agonists

Clonidine and dexmedetomidine are α2-adrenoceptor agonists. Apart from analgesia, they have further effects such as sedation, anxiolysis and sympatholysis, which make them interesting adjuvants in multimodal analgesia regimens [47]. The stimulation of α2-receptors in the dorsal horn of the spinal column, leading to the inhibition of nociceptive neurons and decrease in the release of substance P, is held responsible for their analgesic effect [48]. In addition, they act on presynaptic α2-adrenoceptors in the vasomotor centre of the brainstem. Through activation of these receptors, a negative feedback loop is activated, resulting in a decrease in sympathetic activity [49].

Clonidine and dexmedetomidine have different selectivity for α2-adrenoceptors, with dexmedetomidine being approximately 8-times more specific to alpha-2 adrenoceptors than clonidine [50].

Several studies demonstrated an opioid-sparing and analgesic effect of the perioperative systemic administration of α2-agonists [51–56]. In a systematic review and meta-analysis, Blaudszun et al. investigated the impact of perioperative systemic α2-agonists on postoperative pain severity and morphine usage. They revealed that both clonidine and dexmedetomidine exerted a morphine-sparing effect and lead to a decrease of pain intensity, with the effect being more pronounced for dexmedetomidine than clonidine [53]. At 48 h postoperatively, α2-agonists seemed to have lost their pain-relieving effect. In addition, the incidence of early nausea diminished with both agents with a number needed to treat (NNT) of approximately 9. Although α2-agonists have sedative effects, there was no evidence of a lengthened recovery time. Similar results were seen in a systematic review and meta-analysis, including 57 trials, on clonidine [55]. Although they could not show a reduction in pain scores at rest, clonidine reduced cumulative analgesic consumption at 24 and 36 h, as well as postoperative shivering and PONV. Awakening time was not prolonged.

Apart from the sole analgesic effects, α2-agonists have been used to attenuate hemodynamic stress. Clonidine could be shown to improve hemodynamic stability after tracheal intubation [55], during laparoscopic cholecystectomy [57, 58] and during thyroidectomy surgery [59]. Similar effects could be shown for dexmedetomidine [60]. Furthermore, Wang et al. conducted a study on the effects of dexmedetomidine on inflammation, immune function and perioperative stress in surgical patients [61]. They concluded that dexmedetomidine attenuates perioperative stress and inflammation and preserves immune function.

In summary, perioperative administration of clonidine or dexmedetomidine might be especially beneficial in patients and surgeries, where attenuation of sympathetic stimulation is desired, such as in thyroid or laparoscopic surgery.

Apart from the systemic application, α2-agonists are also useful as an adjunct for regional anesthesia leading to a prolongation of the duration of sensory block and analgesia, as several studies have shown [62–64].

### Adverse Events

The most important perioperative adverse events are hypotension and, especially for dexmedetomidine, bradycardia. In their systematic review, Blaudszun et al. showed that intraoperative and postoperative hypotension was more common after clonidine administration, with a number needed to harm (NNH) of approximately 9 and 20 respectively, whereas there was a higher risk of postoperative bradycardia with dexmedetomidine use with a NNH of 3 [53]. Similar effects could be seen if used as adjuncts for brachial plexus nerve block: dexmedetomidine was associated with bradycardia requiring intervention and both dexmedetomidine and clonidine were associated with hypotension [62]. In 2019, Demiri et al. conducted a study with similar results [65]. The risk of hypotension and bradycardia persisted even after cessation of treatment. However, when looking at dexmedetomidine, a dose-dependency was detected: intraoperative hypotension and postoperative bradycardia were not observed with a bolus dosage of dexmedetomidine of less than 0.5 μg/kg or with continuous administration alone.

Therefore, hemodynamic monitoring is essential when administering clonidine or dexmedetomidine and this must be continued for a prolonged period even after cessation of treatment. The hemodynamic effects must be considered, especially in patients where hypotension and bradycardia might be deleterious, and α2-agonists should be administered with caution in surgeries where a high blood loss is expected.

## Glucocorticoids

The analgesic effects of glucocorticoids are thought to originate from their anti-inflammatory actions, including the suppression of inflammatory cytokines and prostaglandine synthesis as well as induction of anti-inflammatory cytokines. Furthermore, rapid antihyperalgesic effects are believed to be a result of nongenomic effects which reduce the excitability of nerve cells by decreasing glutamate release and increasing the release of γ-aminobutyric acid [66].

Although there are a few trials suggesting that methylprednisolone might have analgesic properties when given perioperatively [67–69], and a recent systematic review and meta-analysis showed similar efficacy of methylprednisolone and dexamethasone in reducing postoperative pain after third molar surgery [70], most studies focused on dexamethasone as a possible adjuvant analgesic in the perioperative setting.

Dexamethasone is commonly used for the prevention of postoperative nausea and vomiting [71], but there is evidence that it has analgesic properties as well. In a meta-analysis of 24 RCTs, dexamethasone in doses larger than 0.1 mg/kg were found to decrease opioid consumption and postoperative pain [72]. Preoperative administration of dexamethasone seemed to provide a greater effect than intraoperative administration. Similar effects could be demonstrated in another systematic review [73]. Waldron et al. showed that a single dose of dexamethasone provided minor yet statistically significant analgesic advantages: patients who were administered dexamethasone exhibited reduced pain scores at 2 and 24 h, along with decreased opioid usage, diminished requirement for rescue analgesia, prolonged time to the first analgesic dose and shorter stays in the post-anesthesia care unit (PACU). In contrast to the study of De Oliveira at el. they showed no dose responsiveness regarding opioid consumption and only a small, probably only minimally clinically significant dose responsiveness regarding pain scores. However, the study is limited by a significant heterogeneity of I^2^ = 94% for VAS pain scores at 2 h and I^2^ = 97% for VAS pain scores at 24 h.

In addition to its analgesic effect when given systemically, dexamethasone can be used to prolong the analgesic duration of peripheral nerve blocks [74]. Pehora et al. showed that both perineural and intravenous dexamethasone led to a prolonged duration of sensory block as well as a reduction in pain intensity at 12 and 24 h postoperatively when given as an adjuvant to peripheral nerve block in upper-limb surgery [75]. At 48 h, no more effect could be detected. Although the duration of block was significantly longer by three hours, and the postoperative pain intensity was significantly lower when dexamethasone was given perineurally compared to intravenously, the mean difference in reduction of postoperative pain intensity did not surpass the predetermined minimally important difference, suggesting the result might not be clinically significant. There was not enough evidence to make a conclusion about dexamethasone adjunct in lower-limb surgery as only two of the included trials reported on that subject.

In contrast to the use as a systemic analgesic, the timing of administration doesn’t seem to be important: Xu et al. compared pre- and postoperative administration of dexamethasone in addition to an interscalene brachial plexus block in shoulder surgery [76]. The mean duration of analgesia, time to first analgesia as well as opioid consumption did not significantly differ between the two groups.

Unlike α2-agonists, there is no risk of hypotension when dexamethasone is used as an adjunct to peripheral nerve blocks, and there is low-quality evidence that dexamethasone might be a superior adjunct compared to dexmedetomidine as it improves the duration of analgesia by a statistically significant increase of approximately 2.5 h more than dexmedetomidine without the risk of hypotension [77].

### Adverse Events

Long-term treatment with corticosteroids is associated with many side effects, such as adrenal insufficiency, hypertension, osteoporosis, delayed wound healing, hyperglycaemia and even diabetes mellitus [78, 79].

Regarding the perioperative use of dexamethasone, there is no evidence of an increased incidence in wound infection and delayed healing, but dexamethasone might elevate blood glucose levels [72, 73, 80]. In a systematic review, Polderman et al. detected an increase in blood glucose levels after dexamethasone administration but no difference in the rate of postoperative wound or systemic infections (Peto odds ratio (OR) 1.01, I^2^ = 27%) [81]. Due to imprecision in trial results, no statement about the effect on delayed wound healing could be made. A recent non-inferiority trial including 8725 participants with and without diabetes mellitus confirmed these results: There was no increased risk of surgical-site infection after administration of 8 mg dexamethasone, not even in patients with diabetes mellitus [82].

Although no serious adverse events could be detected in the studies on perioperative dexamethasone, rare events might have been missed due to the study design. There is evidence that dexamethasone might increase invasion, proliferation, and angiogenesis in glioblastoma-derived orthotopic tumors, which might worsen prognoses of glioblastoma patients [83–85]. Further studies are needed to determine the clinical significance of this potentially dangerous effect of dexamethasone.

## Lidocaine Infusions

Lidocaine is an amide-type local anesthetic. While the principal mechanism of action of lidocaine as a local anesthetic is by blocking the voltage-gated sodium channels and hence blockade of action potential propagation, the analgesic mechanism of intravenous lidocaine remains uncertain. Several mechanisms have been suggested, such as inhibition of voltage-gated sodium-channels, suppression of inflammatory mediators, and modulation of excitatory as well as inhibitory neurotransmission [86].

Several RCTs have studied the analgesic effects of intravenous lidocaine administered perioperatively in different surgical fields, and subsequent systematic reviews and meta-analyses have been conducted with varying results [87–95]. In a systematic review including 16 RCTs, McCarthy et al. found significant reductions in postoperative pain intensity and opioid consumption in open and laparoscopic abdominal surgery as well as in ambulatory surgery, whereas lidocaine had no impact on patients undergoing tonsillectomy, total hip arthroplasty or cardiopulmonary bypass surgery [87]. These results were confirmed by other meta-analyses focusing on abdominal and elective colorectal surgery, respectively [89, 90]. Systemic lidocaine reduced postoperative pain intensity at early time points, although the difference seen by Rollins et al. did not meet the threshold for a clinically relevant difference. While cumulative opioid consumption was reduced in the study conducted by Sun et al. [90], no difference in postoperative opioid requirement was seen in elective colorectal surgery [89]. Apart from the analgesic benefit, a significant reduction in time to defecation and hospital length of stay was seen in both studies. However, an important limitation of these studies was the significant heterogeneity.

In contrast to these results, more recent studies focusing on bariatric surgery detected only little [95] or no benefit [97] of lidocaine infusions administered perioperatively. These different results might be partly explained by different dosing regimens and length of lidocaine infusions. Yang et al. found that only prolonged lidocaine infusions of a duration of more than 24 h provided beneficial effects such as faster time to first defecation, reduced pain scores and reduced hospitalization duration, while neither short nor long term infusions significantly decreased analgesic requirements [93].

Furthermore, some studies explored the impact of lidocaine infusion in spine surgery and breast surgery with conflicting results as well. While the meta-analyses conducted by Licina et al. and Bi et al. showed a significantly reduced postoperative pain intensity up to 48 h after spine surgery and decreased opioid requirements [92, 94], another meta-analysis showed no such effect [95]. One study investigating the effect of lidocaine in breast surgery found that the incidence of chronic postsurgical pain (CPSP) was significantly reduced and pain scores at rest were lower [95], whereas no such effect was shown by another study [91].

The latest Cochrane review on perioperative systemic lidocaine seems to reflect the conflicting results and often low-quality evidence [88]. There was only sparsely significant evidence on lidocaine’s positive effect regarding postoperative pain intensity and secondary outcomes such as opioid requirements and bowel function, and a reduction in pain intensity was only detectable up to four hours postoperatively. The authors concluded that the evidence was insufficient to demonstrate clear improvements in postoperative pain, gastrointestinal recovery, and opioid consumption with intravenous lidocaine. Therefore, further high-quality studies are needed to determine the analgesic effects of systemic lidocaine in the perioperative setting.

### Adverse Events

As intravenous administration of lidocaine poses a risk of toxicity with a possibly catastrophic outcome, consideration of adverse events is important. Adverse events that could be seen include mild headaches, higher incidence of light-headedness and dry mouth [90], dizziness, visual disturbances and a metallic taste [91] and transient sensory disturbance [92]. Of the 68 studies included in the most recent Cochrane review, 50 commented on adverse events. While 23 of these reported no significant adverse events, the adverse events reported in the remaining 27 studies included light-headedness, drowsiness, bradycardia, or perioral numbness [88]. A meta-analysis could not be performed because of the great heterogeneity of the presented data.

Relative contraindications for the administration of intravenous lidocaine, including cardiac disease or conduction block, electrolyte disorders, renal or hepatic disease, seizure or other neurological disorders, as well as pregnancy and breast-feeding should be taken into account [97] [95], and one has to keep in mind that early signs of toxicity might be missed during general anesthesia.

## Others

### Gabapentinoids

Pregabalin and gabapentin have been extensively used in the management of chronic pain conditions and are, beside antidepressants, considered co-analgesics of choice for neuropathic pain [97]. There is little evidence examining the use in the perioperative setting. A systematic review by Tiippana et al. indicates an opioid-sparing effect within the first 24 h post-surgery and a reduction of opioid-related adverse events. However, other trials examining the perioperative use in various surgical populations yielded conflicting results [95, 97, 95]. The use of gabapentinoids was even associated with a greater risk of side effects such as visual disturbances, dizziness and respiratory depression [97]. Based on this evidence, the routine use of gabapentinoids in perioperative pain management cannot be generally recommended.

### Antidepressants

Although antidepressants play an important role in the management of chronic neuropathic pain [95], they are not commonly used perioperatively. Despite a limited number of systematic reviews indicating a favorable effect, including lower postoperative pain scores and reduced opioid consumption, when duloxetine or selective serotonin norepinephrine reuptake inhibitors in general were administered perioperatively [97, 95, 109], overall quality of evidence is considered low because of high interstudy heterogeneity and high risk of bias. Therefore, further high quality randomized controlled trials are needed to support or refute these results.

### Cannabinoids

During the last decades, interest in the medicinal use of cannabinoids has risen, including their use in the management of acute pain. However, evidence is still sparse. While there are a few trials suggesting a beneficial effect [97, 95], other trials showed no [109] or even a negative effect, i.e. an increase in pain scores [97]. A recent systematic review including six trials revealed a small but statistically significant decrease in postoperative pain score with the administration of cannabinoids [95], but overall quality of evidence was considered low. Therefore, the use of cannabinoids in acute pain management must be examined in future high-quality studies.

## Conclusion

In summary, adjuvant analgesics are an important part of multimodal analgesia regimens, especially for painful procedures, as they can reduce postoperative pain intensity as well as opioid requirements.

Ketamine has been proven effective in painful procedures such as thoracic, abdominal, and major orthopedic surgery and might be beneficial in opioid-dependent patients requiring surgery. Besides their analgesic effect, magnesium and α2-agonists can attenuate hemodynamic response, which make them especially useful in laparoscopic procedures. Furthermore, α2-agonists can prolong the analgesic effect when given as an adjunct to regional anesthesia techniques, an effect that could also be shown for dexamethasone, even when administered systemically. While hypotension is an important side effect of α2-agonists, no effect on blood pressure is seen with dexamethasone use, which may make it the more suitable option in patients where hypotension must be avoided. Systemic lidocaine may reduce postoperative pain intensity in abdominal surgery, but evidence remains conflicting.

Although several studies demonstrated the positive effects of various adjuvant analgesics, several unresolved questions persist, awaiting future exploration. There is still uncertainty concerning the optimal dosing of many of adjuvant analgesics in the perioperative setting. In addition, serious adverse events might have been missed due to small sample sizes. Therefore, additional high-quality studies are needed to assess the optimal dosing and the potential risks of the different co-analgesic agents to specify the optimal treatment in different patient groups.

## Data Availability

No datasets were generated or analysed during the current study.

## Abbreviations

CPSP: Chronic postsurgical pain
ERAS: Enhanced recovery after surgery
IASP: International Association for the Study of Pain
NMDA: N-methyl-D-aspartate
NNH: Number needed to harm
NNT: Number needed to treat
PACU: Post-anesthesia care unit
PONV: Postoperative nausea and vomiting
RCT: Randomized controlled trial
VAS: Visual Analogue Scale
